# Translation, Adaptation, and Validation of the Self-Efficacy Scale for Clinical Nurse Leaders for the Portuguese Culture

**DOI:** 10.3390/ijerph19148590

**Published:** 2022-07-14

**Authors:** Marlene Carvalho, Filomena Gaspar, Teresa Potra, Pedro Lucas

**Affiliations:** 1 Nursing Research, Innovation and Development Centre of Lisbon (CIDNUR), Nursing School of Lisbon, 1600-190 Lisbon, Portugal; mfgaspar@esel.pt (F.G.); tsantos@esel.pt (T.P.); prlucas@esel.pt (P.L.); 2 Central Lisbon Hospital and University Centre, 1150-199 Lisbon, Portugal

**Keywords:** health services administration, leadership, management, nurses, psychometric properties, validation studies

## Abstract

Studies related to the competencies of clinical nurse leaders as an advanced practice demonstrate that they are an asset to health organizations. However, it is pertinent to use an instrument to measure the self-efficacy of clinical nurse leaders in Portugal to facilitate managers’ decision-making. In this study, we aimed to translate, adapt, and validate the Clinical Nurse Leader Self-Efficacy Scale for the Portuguese cultural context. This is a quantitative, observational, and descriptive cross-sectional study. The sample of this study was non-probabilistic and intentional, consisting of 329 nurses responsible for shift and specialist nurses. The translation and validation process followed the internationally recognized guidelines on the psychometric properties of measurement instruments. The factor analysis explained 62.1% of the variance and had a seven-dimensional structure. The seven factors were: Patient-Centred Care; Unit Management; Clinical Leadership; Strategic Leadership; Team Management; Cost Reduction; and Care Planning. The confirmatory analysis showed that the seven-factor model achieved a suitable adjustment in the Portuguese context. The self-efficacy scale for clinical nurse leaders can be considered a valid and reliable instrument for application in Portugal in any care context. This scale allows the assessment of the nurse’s perception of their ability to intervene effectively as a clinical leader in their care team.

## 1. Introduction

Policymakers increasingly recognize the need to align the size, composition, skills, and performance of health sector workforces with the increase and diversification of population needs [1]. At the International Workforce Forum in 2019, the reality of the current state of the global nursing workforce was confirmed, warning that actions are needed to promote positive and supportive work environments, including fair funding, safe teams, and professional development [2].

For professional nursing development, it is essential to develop an advanced practice that promotes the quality of nursing care. An advanced nursing practice involves the acquisition of advanced clinical knowledge and skills of greater depth, the complexity of critical thinking, and more autonomous roles to respond to the care needs of individuals, families, groups, and communities. Nurses with advanced practice skills are well positioned to promote evidence-based care practices and exercise effective clinical leadership in both the micro- and the mesosystem [3].

These nurses should develop skills of evaluation, diagnosis, planning, implementation, and evaluation of the necessary care [1,4], in which care should be focused on the nurse–client relationship to achieve positive results, as well as problem-solving and decision-making.

Thus, the leadership of the teams is decisive. The concept of leadership speaks to a multidisciplinary approach to quality patient care, which is conducted by experienced, autonomous, competent, and responsible professionals [4]. Therefore, without the appropriate skills and knowledge, it becomes difficult for nursing leaders to maintain a favourable work environment that promotes quality of care [5]. Here, the intervention of nurse managers is fundamental. A managing nurse is a driver of change towards excellence, through organizing existing resources and creating a safe environment for quality care [5].

Leading nurses influence nursing practice environments and the quality of nursing care [5,6] with their practice and intervention. Nursing practice environments are changeable; that is, the action of the nurse manager is decisive in making them favourable [7]. The nurse, as a leader, is essential to improving communication between teams to achieve goals and increase the quality of nursing care, promote care safety, and develop innovation in practice [5].

Clinical nurse leaders are responsible for their own identity and professional practice, and have essential and crucial characteristics and skills for their role. Within the scope of their autonomy and initiative, they design and implement care, and are responsible for improving nursing outcomes, quality, and the cost-benefit ratio [3]. Clinical nurse leaders’ competencies include compassion, intelligence, and confidence [8,9,10,11]. Intelligence and the ability to work with others under stress are necessary characteristics, as their workload can be adjusted at any given moment, as they are a change agent and care coordinator [12]. The measures implemented by the clinical nurse leader must be agreed on by the other team members [12]. The clinical nurse leader should be an element of advanced practice and be motivated to perform their functions [12].

In the literature review we conducted, Carvalho and Lucas (2020) [3] demonstrated that clinical nurse leaders work at the level of services and units and are responsible for coordination between disciplines and the management of clinical results, with a special focus on health promotion and disease prevention in patients and the implementation of programs for quality improvement and clinical risk management [3,13]. They have a vision for the role and measurement of the impact of care, as well as critical thinking and skills for reviewing client outcomes and teaching/managing change and evidence-based practices [3,12]. Thus, they boost quality and are active [3,8,9,10,11,14].

Therefore, the leading clinical nurse:

- Integrates the management of the units, promoting a calm and structured care environment through the coordination of the team and strategies for change [8,9,10,11,15];
- Is an information manager who uses the research results and promotes the fluidity of communication in clinical practice [3];
- Is a team leader, information manager, and systems analyst and anticipates clinical risks, alongside being a provider of excellence in all care delivery contexts [3,8,9,10,11,16];
- Advocates for the integration of different disciplines in the provision of care, improves safety, and demonstrates critical thinking, communication, and strong assessment skills [17];
- Knows information systems and standardized languages to improve clinical outcomes, participates in monitoring of the performance of their team, and supports evidence-based decision-making and practice [10,11];
- Actively intervenes in improving the quality of care provided to patients, based on evidence-based practice and critical thinking [10,11].

The clinical nurse leader practices transformational leadership, an exemplary professional practice, and implementation of new knowledge [18,19]. Thus, the evidence also reports capacity for clinical leadership, team leadership, information management, and resource management, and adds the defense not only of patients but of communities and health programs, presenting a potential for practice in new environments [14,18,19].

In short, it is an advanced practice nurse who develops the critical thinking and evaluation skills essential to promoting evidence-based practice. Subsequently, they use evidence to support decision-making in relation to practice, as well as to influence team and organizational practices [11,13]. Thus, the core practice competencies of clinical nurse leaders can be organized into three domains: (a) nursing leadership in care management and coordination; (b) clinical outcome management to promote evidence-based practice and decision-making; and (c) efficient management to promote quality of nursing care and client safety [20,21].

Gilmartin states that self-confidence is a significant predictor of successful career transitions [21]. The confidence of co-workers and managers in clinical nurse leaders becomes crucial to their ability to perform their roles effectively, notably in promoting overall team performance, job satisfaction, and retention of nurses [18]. Clinical nurse leaders with more developed self-efficacy have improved performances [3,21].

Therefore, self-efficacy is important to successful role transition, job satisfaction, and performance [3]. The Self-Efficacy Assessment Scale of Clinical Nurse Leaders by Gilmartin and Nokes assesses nurses’ perception of their ability to intervene effectively in the performance of their clinical leadership functions [3,20]. Assessing the self-efficacy of leading clinical nurses throughout their practice is fundamental for the provision of care and the management of health units and organizations, as they improve the performance of these professionals, increasing the quality and safety of nursing care and health gains [3].

Function transitions imply two interdependent adjustment processes: personal development and role development [21]. This transition process is increasingly complex due to the increased demands on the practices of clinical nurse leaders. Hiring clinical nurse leaders is an effective approach towards ensuring excellent nursing care, maximizing research, and influencing the ways other health professions provide care in the microsystem. This transition is an ongoing process in which age, academic degree, and professional experience can be catalysts for change and wisdom [18,19,20,21].

Currently, greater emphasis is placed on evidence-based practice to support clinical decision-making. This decision is supported by the best evidence, based on nurses’ experience, client values, and quality care needs, as well as care costs [22].

Likewise, the role developed by the clinical nurse leader focuses on the management of patient-centred care and clinical excellence [21]. Thus, it is pertinent to evaluate the effectiveness of the clinical nurse leader in their care team. Given the lack of a validated scale for the Portuguese population, this study performed the cultural and linguistic adaptation of the Self-Efficacy Scale for Clinical Nurse Leaders after authorization from the original author. This is the first psychometric validation study of this scale outside the United States of America.

In this study, we aimed to translate, adapt, and psychometrically validate the Self-Efficacy Scale for Clinical Nurse Leaders for the Portuguese cultural context (SESCNL-PT), starting from the guiding research question: to what extent does the SESCNL-PT have psychometric qualities suitable for the Portuguese cultural context?

## 2. Materials and Methods

### 2.1. Study Design

This is a quantitative, observational, and descriptive cross-sectional study of the translation and adaptation of the SESCNL scale to the Portuguese cultural context, and its psychometric validation.

Gilmartin and Nokes’ (2015) original scale [20] assessed nurses’ perceptions of their abilities to function effectively as a clinical nurse leader, and it demonstrated promising measurement properties. The discriminant validity was assessed by examining inter-correlations among the indices. The inter-correlations among the indices range between 0.72 and 0.46. The reliability of the scale was examined, assessing the internal consistency of CNLSES by Cronbach’s alpha scores between 0.83 and 0.98.

### 2.2. Methods

The development of the linguistic and conceptual equivalence of the SESCNL-PT followed the methodological guidelines of several authors [22,23,24,25,26,27], namely Beaton et al. (2000) and Sousa and Rojjanasrirat (2011) [25,26]. The translation of the original English version into Portuguese was prepared independently by two Portuguese and bilingual translators. One bilingual, fluent in Portuguese and English, with knowledge in the nursing area and another translator with no knowledge in the health area. The analysis of the discrepancies between the two versions resulted in a consensual translation version in the target language, which was subjected to retroversion by two translators of English origin. After analysis of the two versions, the instrument was adjusted to obtain the semantic, idiomatic, and conceptual equivalence of the items. The conceptual equivalence focuses on determining whether people from two different cultures of the original and translated scale identify the concept/variable from the same perspective, so that the concept is assessed as the same [22]. Semantic equivalence focuses on the meaning of each item; whether the meaning of the words/phrases in the translation remains the same and is intelligible to the respondents [22]. In the next stage, the analysis was submitted to a group of experts (nurses responsible for the shift and with clinical specialty) with characteristics similar to those of the population under study and, finally, in the third phase, the study proceeded to psychometric validation of the instrument.

A group of 22 clinical nurse leaders from a hospital in the Lisbon region received the reliability evaluation, or test–retest, at two separate times, about two weeks apart, in March 2018. The analysis evaluated the clarity and degree of understanding of the questions, as well as the cultural relevance. The questionnaire was delivered along with an envelope to be returned closed and anonymized.

The translated questionnaire was evaluated by eight clinical nurse leaders working in hospitals with extensive clinical experience, fluent in English and Portuguese, with work experience in the Portuguese health system for at least the last five years, and familiarity with research processes. They were asked to assess whether the version was easy to understand, whether it was adapted to the Portuguese context, and whether it could be used in Portugal.

The analysis of the scale items allowed us to verify the absence of missing data and confirmed all questions were answered. Pearson’s correlation coefficient was 0.77 both times. Studies have determined that values below 0.70 are considered satisfactory [27,28], verifying a reasonable consistency [24].

#### 2.2.1. Data Collection and Procedures

The data collection was conducted in a hospital centre in the Lisbon region between September and December 2018, and the treatment took place in 2019, after authorization from the Board of Directors and the Ethics Committee.

The data collection instrument was presented with information about its purpose and instructions on the cover page. The questionnaire was composed of three parts: Part A—general characterization, sociodemographic and professional characterization; Part B—clinical leadership, with the translated version consisting of 56 items evaluating the effectiveness of the clinical nurse leader, each item rated on a five-point Likert-type confidence scale; and Part C—suggestions.

#### 2.2.2. Sample

The sample of this study was non-probabilistic and intentional [24]. The inclusion criteria for the selection of the sample were: nurses who had been shift managers over their professional careers, with three fundamental functions/responsibilities: planning, coordination, and evaluation of nursing activities; nurses with a postgraduate degree in an area of specialization, performing in specialist positions for more than six months; shift managers and/or postgraduate degree nurses in an area of specialization able to perform tasks in a hospital context, over more than six months; shift managers and/or postgraduate degree nurses in an area of specialization who agreed to participate in the study; inpatient units in which the managing nurses were allowed to participate in the study; in a hospital in the Lisbon region with a highly scientific, technical, and technological differentiation, and one considered a Portuguese reference institution.

We gave out 612 questionnaires and received a response from 329 nurses for this validation study. This sample size is in line with the authors’ suggestions for validation studies [28].

### 2.3. Analysis

IBM^®^ SPSS^®^ Software and AMOS Software version 25 (IBM Corp, Armonk, NY, USA) were used to analyze the data to evaluate the psychometric qualities of SESCNL. The criterion validity established the comparison between the proposed measure and another measure that served as a criterion, and it assessed the sensitivity for the same [27].

Factor analysis evaluated the construct validity through analysis of the main components.

The study extracted the main components using the Varimax rotation method when performing the exploratory factor analysis. In the investigation of data adequacy of the exploratory factor analysis, the study used the Kaiser-Meyer-Olkin test of sampling adequacy measurement (KMO) method and Bartlett’s sphericity test, whose value should be greater than 0.5 [24,28]. The analysis always used the cut-off point of 0.4; that is, variables that assumed correlation values equal to or greater than 0.4 were part of the dimension [24]. The research analyzed the total explained variance of each dimension.

The reliability and internal consistency of the dimensions and the instrument were estimated from Cronbach’s alpha internal consistency measure in the seven dimensions, considering an acceptable reliability if α ≥ 0.70 [27].

The study evaluated the overall adequacy of the model in the confirmatory factor analysis (CFA), using as indices of the quality of the adjustment of the SESCNL model: Chi-square adjustment test statistics (DHL 2 /df), CFI, GFI, RMSEA, and MECVI. Important considerations before starting CFA were the guarantee of multivariate normality: no variable presented values of Sk and Ku indicators of severe violation of the normal distribution (|Sk| < 3 and |Ku| < 10), and at least two factors had at least two indicators per factor and the presence of outliers: the Mahalanobis square distance (D2) [28].

### 2.4. Ethical Considerations

The investigation process respected ethical and legal principles and was approved by the Board of Directors and Ethics Committee with the number 588/2018. The free and informed consent of the participants in the study was guaranteed, according to Gray, Grove and Sutherland [22]. The study ensured the confidentiality and anonymity of the participants. The data were stored and treated anonymously and confidentially.

The translation and adaptation of the scale for the Portuguese cultural context had the authorization of the author of the original scale, which can be found in Supplementary Materials.

## 3. Results

A total of 612 questionnaires were submitted, and the sample consisted of 329 nurses, corresponding to a response rate of 53.8%.

Of the sample, 80.9% of the participants were female. For academic qualifications, predominant was a degree, at 75.4%.

Regarding age group, the majority were between 34 and 39 years (23%) or between 44 and 49 years (20%). The mean age was 41.5 years old; the sample was predominantly young/adult. The minimum age was 24 years, and the maximum was 61 years.

This sample represented participants without the title of specialist nurse (51.7%). Of those holding a specialist nurse title (48.3%), the most typical specialties were Rehabilitation (15.5%), Child and Paediatric Health (13.7%), and Maternal Health and Obstetric (8.5%). For professional activity experience, most had been in the profession for 11–16 years (85%), closely followed by those in the profession for 21–26 years (79%). The maximum time was 30 years, and the average was 11 years. Within the scope of professional activity in the institution, the research confirmed a uniform distribution, especially for the period of 7 to 12 years (26.1%) and 22 to 27 years (20.4%).

### 3.1. Exploratory Factor Analysis

At the end of the analysis, the study eliminated the items 5, 11, 19, 23, 24, 29, 30, 33, and 54 due to their presenting factor weights below 0.40 [24].

The final questionnaire consisted of 47 items in 7 final components: patient-centred care (11 items), unit management (10 items), clinical leadership (8 items), strategic leadership (5 items), team management (7 items), cost reduction (4 items), and care planning (2 items).

After analyzing the factorial loads by components, determining the KMO and Bartlett’s sphericity test, the study verified that the KMO presented a value of 0.94, *p*-value < 0.001, which indicated an excellent correlation [24] between the SESCNL-PT variables. Bartlett’s sphericity test demonstrated a *p*-value < 0.001 with a significance of less than 0.05, demonstrating that the variables were significantly correlated, and this sample was suitable for analysis. Table 1 shows that the study obtained results with a 7-factor matrix, which explains 62.1% of the variance of the phenomenon under study, with an eigenvalue of 1.246.

The components of the scale were as follows:

Component 1: Patient-Centred Care—refers to patient-centred care practices based on scientific evidence, using information systems. This dimension also explores the unit and its performance, given the quality of care and patient safety. It includes items 1, 2, 6, 7, 8, 9, 10, 12, 13, 14, and 15.

Component 2: Unit Management—reports to clinical supervision, where it aims to promote autonomous decision-making, valuing client protection, safety, and quality of care in the integration of nurses, and the guidance of nurses in training in clinical education. This dimension also includes information about the importance of continuous practice and the availability of a team/service/organization for its completion. It includes items 32, 34, 35, 44, 45, 46, 47, 48, 49, and 50.

Component 3: Clinical Leadership—related to organizational leadership since it refers to a set of actions, such as assertive choice of strategies, organization of activities, motivation of employees, maintenance of interpersonal relationships, development of skills and competencies, and crisis management. It includes items 36, 37, 38, 39, 40, 41, 42, and 43.

Component 4: Strategic Leadership—part of the alignment between the organization’s strategic objectives, culture, perception of organization gaps, competencies, and leadership systems. These factors must be fully aligned for maximum effectiveness in the outcome of care. It includes items 51, 52, 53, 55, and 56.

Component 5: Team Management—the ability to perform good team management is fundamental to success. A team manager nurse should be able to lead, motivate, inspire, support, and believe in the potential of their team, and be seen as an inspiring element that represents the values of the organization and is committed to achieving the best results for patient care. It includes items 16, 17, 18, 20, 21, 22, and 25.

Component 6: Cost Reduction—related to the improvement of the organization of nursing care methods. Thus, weaknesses and unnecessary waste are detected that can be reduced or eliminated, creating opportunities for cost reduction. It includes items 26, 27, 28, and 31.

Component 7: Care Planning—involves the responsibility for the well-being of the client, identifying the needs, and involving the client in the elaboration/endorsement of the care plan, with the client as the central focus of care. It includes items 3 and 4.

### 3.2. Reliability Analysis

The reliability of the Self-Efficacy Scale for Clinical Nurse Leaders for the Portuguese cultural context was α = 0.96, which is an excellent value [24]. The dimension of Clinic Leadership had very good consistency (α = 0.91), and the other dimensions of Patient-Centred Care (α = 0,90), Unit Management (α = 0,89), Strategic Leadership (α = 0.86), Team Management (α = 0,88), Cost Reduction (α = 0,84), and Care Planning (α = 0,82) were considered good [24].

### 3.3. Confirmatory Factor Analysis

The septa-factorial model of SESCNL-PT for the Portuguese population revealed an adjustment quality with the need to correct trajectories in the model between the residues of the item pairs e42–e43, (“Cost Reduction”), e37–e39, e36–e37, e40–e41 (“Team Management”), e30–e31 (“Strategic Leadership”), e27–e28, e24–e25, e22–e23 (“Clinical Leadership”), e19–e20, e17–e18, e13–e14 (“Unit Management”), and e10–e11, e6–e7, e3–e5 (“Patient-Centred Care”). Thus, a good adjustment quality (1995.475/999 = 1.997) was obtained with RMSEA = 0.055; P [rmsea ≤ 0.05] < 0.001; MECVI = 7.005) for Standardized RMR values: 0.0723. Although the model was better adjusted, new covariances were performed between e3–e4 (“Patient-Centred Care”). Thus, the research obtained a good adjustment quality (with equal value 1973.461/998 = 1.997 and improved quality adjustment (GFI = 0.800) and Good for CFI = 0.899. The substantial value of RMSEA = 0.055; P [rmsea ≤ 0.05] < 0.001; MECVI = 6.945) was maintained for Standardized RMR values: 0.0721.

**Figure 1:**
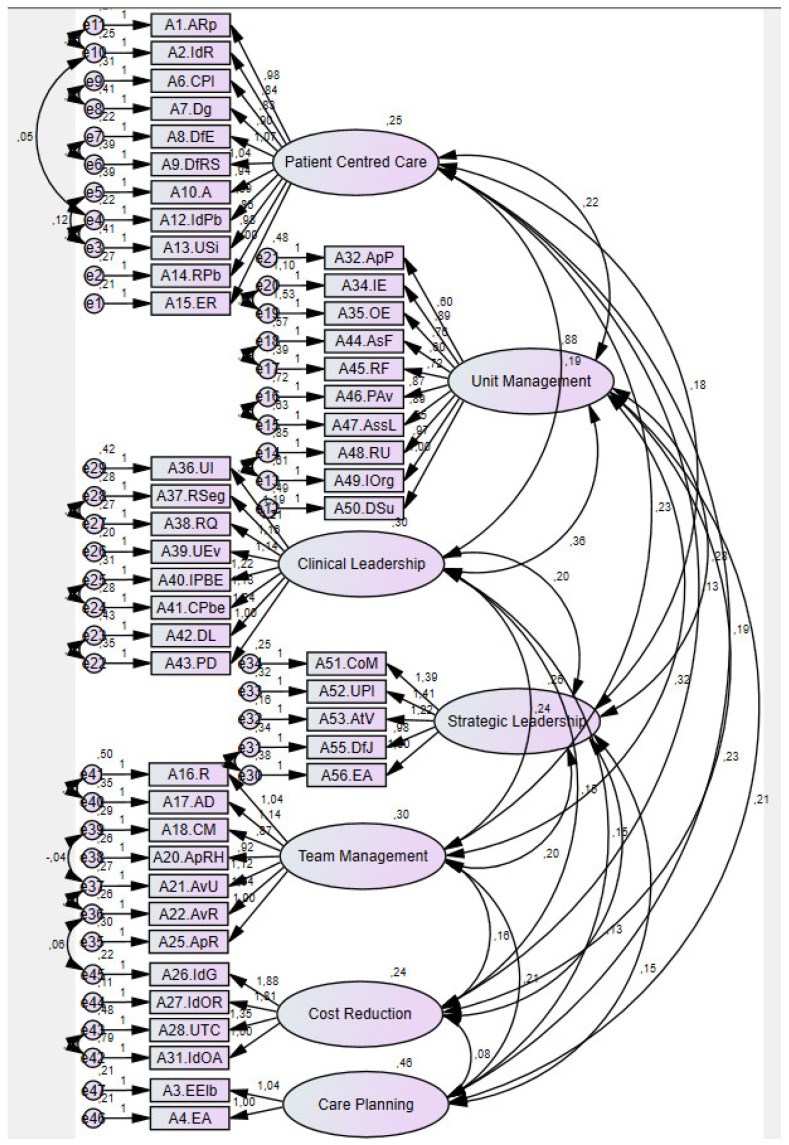
Septa-factorial model of SESCNL-PT for the Portuguese population.

In Figure 1, the septa-factorial model of SESCNL-PT, adjusted to a sample of 329 clinical nurse leaders, presented a good adjustment quality (1919,451/994) = 1931, *p* < 0.05) as well as MECVI parsimony indices (8980), P [rmsea ≤ 0.05] < 0.001 for standardized RMR values: 0.0709. An adjustment of GFI = 0.805 and CFI = 0.904 was obtained.

## 4. Discussion

In this study, we carried out the translation and psychometric validation for the Portuguese population of the Self-Efficacy Scale for Clinical Nurse Leaders, which was the first validation of this scale outside the United States of America. In Portugal, it is the first study of clinical nurse leaders and, therefore, the verification of a measurement instrument with the same theme. For these two reasons, it is a significant study both at the national and international levels.

The results showed that the data collection instrument has excellent reliability.

The questionnaire validated for the Portuguese context ended up with 47 items, in 7 final components.

Some items validated both in Portugal and in the USA were eliminated. The ones that coincided were 19, 29, 30, and 54. In the validation of this study, five more items were removed: 5, 11, 23, 24, and 33.

There were similarities between this study and that of Gilmartin and Nokes (2015) concerning the dimensions.

The septa-factorial model of SESCNL-PT in the Portuguese cultural context presented a good adjustment quality, which was significantly higher than that of the original model (1919.451/994) = 1931, *p* < 0.05 vs. 2675.916/1013 = 2.642), as well as considerably lower parsimony indices MECVI (6.809 vs. 8.980), P [rmsea≤ 0.05] < 0.001 for Standardized RMR values: 0.0709 vs. 0.0723. A significantly improved quality adjustment was obtained with GFI = 0.805 and CFI = 0.904.

Thus, the seven-factor model was the most stable and significantly represented the study sample in Portugal. All coefficients were meaningful, and the model adjustment coefficients were good, supporting the septa-factorial structure for SESCNL-PT.

### Limitations

A limitation of the study was that the questionnaire was applied to a hospital centre, although it was referenced at a national level. It would be helpful to use this scale at the national level in different contexts of nursing practice: in the hospital context, primary healthcare, long-term care, and nursing homes.

Another limitation of the study was the delay in publishing these results, which was due to the research project being associated with a master’s dissertation. We believe that this delay had no influence on the results or the importance of the psychometric validation study.

## 5. Conclusions

The results of the adaptation and validation indicated that SESCNL-PT can be used in the Portuguese cultural context because it systematically followed all methodological steps and achieved satisfactory results regarding its reliability and validity.

The Portuguese version presents adequate sensitivity, reliability, and factor validity, and it can be considered an instrument for the management of patient-centred care and clinical excellence.

The adjustment of the factorial model allows us to propose a version of the scale in Portuguese—the Self-Efficacy Scale for Clinical Nurse Leaders (Escala de Autoeficácia para Enfermeiros Líderes Clínicos–versão portuguesa)—and facilitate an adjustment of the performance of these professionals. Although advanced practice nurses were confident that they positively influence their peers’ practice, there is a lack of evidence that demonstrates their impact. This scale allows for the evaluation of the perception of nurses in the performance of the skills associated with their role, thereby closing this gap.

As implications for the context of practice, this study offers nurse managers a tool that, when applied to practice nurses, provides results that indicate the effectiveness of their clinical leadership; in other words, the essential procedures that nurses in charge of shifts perform and are not recognized as such, although they are crucial in terms of outcomes for patients and consequently for gains in health. This will be a significant contribution to the promotion of favourable nursing practice environments.

In terms of implications for research, this study makes available to the scientific community a data collection instrument validated for the Portuguese language and cultural context. The results of this study allow the establishment of a benchmark with the results of international studies, and place Portuguese nurses in discussion with global researchers on current and critical issues for the valorization and qualification of health systems.

We suggest the development of this theme by applying the SESCNL-PT in hospital settings, in primary healthcare, in long-term care units, and in nursing homes. It is important to develop longitudinal studies with the SESCNL-PT, associated with the measurement of favourable nursing practice environments. Longitudinal studies would allow causal links to be established between CNL and better outcomes for patients, for teams, for the organization and quality of nursing care and for the efficiency of healthcare organizations.

The topic of clinical nurse leaders’ self-efficacy should be addressed in the training of advanced practice nurses and in postgraduate and master’s training on nursing management, because better self-efficacy means better performance by these specialized professionals.

## Figures and Tables

**Table 1:** SESCNL-PT components.

ITEMS	COMPONENTS						
	Patient-Centred Care	Unit Management	Clinical Leadership	Strategic Leadership	Team Management	Cost Reduction	Care Planning
1	0.63						
2	0.61						
6	0.60						
7	0.66						
8	0.73						
9	0.66						
10	0.64						
12	0.57						
13	0.54						
14	0.42						
15	0.57						
32		0.42					
34		0.55					
35		0.45					
44		0.48					
45		0.64					
46		0.74					
47		0.68					
48		0.76					
49		0.80					
50		0.78					
36			0.70				
37			0.66				
38			0.69				
39			0.70				
40			0.68				
41			0.65				
42			0.50				
43			0.44				
51				0.71			
52				0.59			
53				0.74			
55				0.62			
56				0.62			
16					0.50		
17					0.52		
18					0.54		
20					0.53		
21					0.56		
22					0.55		
25					0.43		
26						0.82	
27						0.84	
28						0.59	
31						0.43	
3							0.77
4							0.74
Cronbach’s alpha	0.90	0.89	0.91	0.86	0.88	0.84	0.82

## Data Availability

Restrictions apply to the availability of these data. Data were obtained from a third party and are available with the permission of the third party.

## Supplementary Materials

The following supporting information can be downloaded at: https://www.mdpi.com/article/10.3390/ijerph19148590/s1, a questionnaire.
