# Supplementary material for: Translation, Adaptation, and Validation of the Self-Efficacy Scale for Clinical Nurse Leaders for the Portuguese Culture
Source: Int J Environ Res Public Health. 2022 Jul 14;19(14):8590. doi: 10.3390/ijerph19148590 (PMC9325131; doi:10.3390/ijerph19148590)
Supplement: Supplementary file 1 [file ijerph-19-08590-s001.zip › ijerph-1773919-supplementary.pdf]

In your practice as a Clinical Nurse Leader, how confident are you that you can:					
1. Assume accountability for the welfare of client populations served in your unit?	1	2	3	4	5
2. Identify client population risks based on a comprehensive assessment?	1	2	3	4	5
3. Collaborate with cohorts of clients in designing a total care plan?	1	2	3	4	5
4. Collaborate with clients in gaining their endorsement for the total care plan?	1	2	3	4	5
5. Consult appropriately with other health professionals to design a total plan of care your clients?	1	2	3	4	5
6. Communicate a total plan for clients with other members of the intervention team?	1	2	3	4	5
7. Delegate aspects of a total plan of care to other members of the intervention team?	1	2	3	4	5
8. Advocate effectively on behalf of the client with the intervention team?	1	2	3	4	5
9. Advocate on behalf of the client with the client's network?	1	2	3	4	5
10. Acquire information about the population through information systems?	1	2	3	4	5
11. Seek knowledge about specific populations from the research literature?	1	2	3	4	5
12. Identify population-level health problems?	1	2	3	4	5
13. Use information systems to track population level clinical outcomes?	1	2	3	4	5
14. Resolve population-level health problems?	1	2	3	4	5
15. Engage the intervention team in evaluating progress in achieving desired clinical outcomes?	1	2	3	4	5
16. Meet regularly with the intervention team?	1	2	3	4	5
17. Evaluate the intervention team's performance with achieving patient care outcome goals?	1	2	3	4	5
18. Communicate changes in clients' care plan with the members of the intervention team?	1	2	3	4	5
19. Share knowledge from the literature with other members of the intervention team to improve care?	1	2	3	4	5
20. Appropriately deploy human resources to improve outcomes?	1	2	3	4	5
21. Evaluate how your unit fits with the work of the larger organization?	1	2	3	4	5
22. Evaluate the capacity of resources available to your unit to accomplish its work?	1	2	3	4	5
23. Mobilize managers to deploy resources?	1	2	3	4	5
24. Mobilize informal leaders to deploy resources?	1	2	3	4	5
25. Deploy unit resources effectively to improve aggregate clinical outcomes?	1	2	3	4	5
26. Identify waste in your unit?	1	2	3	4	5
27. Identify opportunities for cost savings in your unit?	1	2	3	4	5
28. Use technology to reduce costs?	1	2	3	4	5
29. Use technology to enhance clinical outcomes?	1	2	3	4	5
30. Set priorities to work efficiently without compromising quality?	1	2	3	4	5
31. Identify opportunities for revenue enhancement to benefit clients?	1	2	3	4	5
32. Create proposals to modify your unit using alternative business models?	1	2	3	4	5
33. Create proposals to modify your unit incorporating return on investment analyses?	1	2	3	4	5
34. Mentor other CNLs?	1	2	3	4	5
35. Act as a preceptor for other CNLs?	1	2	3	4	5
36. Translate clinical research to improve clinical practice routines?	1	2	3	4	5
37. Review your unit's performance to assess risk to client safety?	1	2	3	4	5
38. Review your unit's performance to assess risks to the quality of care?	1	2	3	4	5
39. Use evidence to challenge existing clinical practices?	1	2	3	4	5
40. Incorporate evidence-based practice changes into clinical information systems?	1	2	3	4	5
41. Communicate evidence-based practice modifications to other health professionals?	1	2	3	4	5
42. Provide clinical leadership within your unit?	1	2	3	4	5
43. Promote the professional development of the team members?	1	2	3	4	5
44. Assure the continuing education of the team members?	1	2	3	4	5
45. Educate your unit's staff on innovative practices?	1	2	3	4	5
46. Participate in the performance evaluation of intervention team members?	1	2	3	4	5
47. Assume leadership in organizational governance activities?	1	2	3	4	5
48. Represent your unit on organizational committees?	1	2	3	4	5
49. Act as a leader in relevant professional organizations?	1	2	3	4	5
50. Disseminate your unit's successes in care management to the larger nursing community?	1	2	3	4	5
51. Know the organization's mission?	1	2	3	4	5
52. Apply the organization's strategic plan to guide practice on your unit?	1	2	3	4	5
53. Practice in accordance with the values of the organization?	1	2	3	4	5
54. Participate in the development of the organization's strategic plan?	1	2	3	4	5
55. Advocate for social justice in your unit's activities?	1	2	3	4	5
56. Engage in professional development activities?	1	2	3	4	5
